# Supplementary material for: MRI Delta Radiomics to Track Early Changes in Tumor Following Radiation: Application in Glioblastoma Mouse Model
Source: Biomedicines. 2025 Mar 28;13(4):815. doi: 10.3390/biomedicines13040815 (PMC12024708; doi:10.3390/biomedicines13040815)
Supplement: Supplementary file 1 [file biomedicines-13-00815-s001.zip › biomedicines-3510917-supplementary.docx]

Article

MRI Delta Radiomics to Track Early Changes in Tumor
Following Radiation: Application in Glioblastoma Mouse Model

Mohammed S. Alshuhri ^1,^*, Haitham F. Al-Mubarak ^2^, Abdulrahman Qaisi ^3^, Ahmad A. Alhulail ^1^,
Abdullah G. M. AlMansour ^1^, Yahia Madkhali ^4^, Sahal Alotaibi ^5,6^, Manal Aljuhani ^1^, Othman I. Alomair ^7^,
A. Almudayni ^1^ and F. Alablani ^1^

^1^ Radiology and Medical Imaging Department, College of Applied Medical Sciences, Prince Sattam
Bin Abdulaziz University, Alkharj 11942, Saudi Arabia; a.alhulail@psau.edu.sa (A.A.A.); ag.alqahtani@psau.edu.sa (A.G.M.A.); m.aljuhani@psau.edu.sa (M.A.); a.almudayni@psau.edu.sa (A.A.); f.alablani@psau.edu.sa (F.A.)

^2^ Department of Radiology, Northwestern University, Chicago, IL 60611, USA;
haitham.almubarak@northwestern.edu

^3^ Department of Radiology, Security Forces Hospital, Riyadh 11564, Saudi Arabia; ahq1401@gmail.com

^4^ Department of Diagnostic Radiography Technology, College of Nursing and Health Sciences,
Jazan University, Jazan 45142, Saudi Arabia; ymedkhali@jazanu.edu.sa

^5^ Radiological Sciences Department, College of Applied Medical Sciences, Taif University,
Taif 21944, Saudi Arabia; dahhasi@gmail.com

^6^ Faculty of Health & Life Sciences, University of Liverpool, Liverpool L69 7ZA, UK

^7^ Radiological Sciences Department, College of Applied Medical Sciences, King Saud University,
P.O. Box 145111, Riyadh 4545, Saudi Arabia; oalomir@ksu.edu.sa

***** Correspondence: m.alshuhri@psau.edu.sa

**Table S1.** The robust radiomic features demonstrate high inter-observer reliability, with Intraclass Correlation Coefficients (ICCs) exceeding 0.8.

| **No.** | Features Names | category |
| --- | --- | --- |
| 1 | Elongation | shape |
| 2 | Flatness | shape |
| 3 | LeastAxisLength | shape |
| 4 | Maximum2DDiameterColumn | shape |
| 5 | Maximum2DDiameterRow | shape |
| 6 | Maximum3DDiameter | shape |
| 7 | MeshVolume | shape |
| 8 | MinorAxisLength | shape |
| 9 | SurfaceArea | shape |
| 10 | SurfaceVolumeRatio | shape |
| 11 | VoxelVolume | shape |
| 12 | x10Percentile | histogram |
| 13 | x90Percentile | histogram |
| 14 | Energy | histogram |
| 15 | Entropy | histogram |
| 16 | InterquartileRange | histogram |
| 17 | MeanAbsoluteDeviation | histogram |
| 18 | Mean | histogram |
| 19 | Median | histogram |
| 20 | Minimum | histogram |
| 21 | RobustMeanAbsoluteDeviation | histogram |
| 22 | RootMeanSquared | histogram |
| 23 | Skewness | histogram |
| 24 | TotalEnergy | histogram |
| 25 | ClusterProminence | GLCM |
| 26 | ClusterShade | GLCM |
| 27 | ClusterTendency | GLCM |
| 28 | Contrast | GLCM |
| 29 | Correlation | GLCM |
| 30 | DifferenceAverage | GLCM |
| 31 | DifferenceEntropy | GLCM |
| 32 | DifferenceVariance | GLCM |
| 33 | Imc1 | GLCM |
| 34 | Imc2 | GLCM |
| 35 | InverseVariance | GLCM |
| 36 | JointEnergy | GLCM |
| 37 | JointEntropy | GLCM |
| 38 | SumEntropy | GLCM |
| 39 | SumSquares | GLCM |
| 40 | DependenceNonUniformity | GLDM |
| 41 | GrayLevelNonUniformity | GLDM |
| 42 | GrayLevelVariance | GLDM |
| 43 | LargeDependenceEmphasis | GLDM |
| 44 | SmallDependenceEmphasis | GLDM |
| 45 | SmallDependenceHighGrayLevelEmphasis | GLDM |
| 46 | GrayLevelNonUniformity_1 | GLRLM |
| 47 | GrayLevelVariance_1 | GLRLM |
| 48 | LongRunEmphasis | GLRLM |
| 49 | RunEntropy | GLRLM |
| 50 | RunPercentage | GLRLM |
| 51 | RunVariance | GLRLM |
| 52 | ShortRunEmphasis | GLRLM |
| 53 | ShortRunHighGrayLevelEmphasis | GLRLM |
| 54 | LargeAreaEmphasis | GLSZM |
| 55 | LargeAreaLowGrayLevelEmphasis | GLSZM |
| 56 | Busyness | NGTDM |
| 57 | Complexity | NGTDM |
| 58 | Contrast_1 | NGTDM |
